# Nitrogen recycling from the xylem in rice leaves: dependence upon metabolism and associated changes in xylem hydraulics

**DOI:** 10.1093/jxb/erw132

**Published:** 2016-04-06

**Authors:** Karen J. Bailey, Richard C. Leegood

**Affiliations:** Robert Hill Institute and Department of Animal and Plant Sciences, University of Sheffield, Sheffield S10 2TN, UK

**Keywords:** Amides, amino acids, asparagine, hydathode, phospho*enol*pyruvate carboxykinase, rice (*Oryza sativa*), xylem.

## Abstract

Measurements of amino acids in the guttation fluid and in the xylem exudates of cut leaves from intact plants provide evidence of the remarkable efficiency with which these nitrogenous compounds are reabsorbed from the xylem sap. This could be achieved by mechanisms involving intercellular transport and/or metabolism. Developmental changes in transcripts and protein showed that transcripts for phospho*enol*pyruvate carboxykinase (PEPCK) increased from the base to the leaf tip, and were markedly increased by supplying asparagine. Supplying amino acids also increased the amounts of protein of PEPCK and, to a lesser extent, of pyruvate, Pi dikinase. PEPCK is present in the hydathodes, stomata and vascular parenchyma of rice leaves. Evidence for the role of PEPCK was obtained by using 3-mercaptopicolinic acid (MPA), a specific inhibitor of PEPCK, and by using an activation-tagged rice line that had an increase in PEPCK activity, to show that activation of PEPCK resulted in a decrease in N in the guttation fluid and that treatment by MPA resulted in an increase in amino acids in the guttation fluid and xylem sap towards the leaf tip. Furthermore, increasing PEPCK activity decreased the amount of guttation fluid, whereas decreasing PEPCK activity increased the amount of xylem sap or guttation fluid towards the leaf tip. The findings suggest the following hypotheses: (i) both metabolism and transport are involved in xylem recycling and (ii) excess N is the signal involved in modulating xylem hydraulics, perhaps via nutrient regulation of water-transporting aquaporins. Water relations and vascular metabolism and transport are thus intimately linked.

## Highlight

Manipulation of phospho*enol*pyruvate carboxykinase activity in rice leaves suggests that metabolism and transport participate in recycling of xylem amino acids and amides and that excess N modulates xylem hydraulics.

## Introduction

The xylem not only supplies the aerial parts of the plant with water, but also transports nutrients from the root to the shoot. These include inorganic and organic forms of nitrogen, amino acids and amides. Therefore, there is a major flux of these solutes from the roots to the leaves via the transpiration stream. Since the xylem supplies both sources and sinks, it is unlikely that it always supplies the nitrogenous compounds that are required. For example, in the developing leaves of grasses, the developing sink tissues are at the base of the leaf and mature source tissues at the leaf tip. In the sink tissues of the leaf, it is probable that the majority of imported nitrogen is used to make proteins for photosynthesis and other processes, or for storage, whereas in the developed source tissues the potential input of nitrogen and other solutes via the transpiration stream is probably in excess of what is needed for biosynthesis. In these source tissues amino acids are recycled from the xylem into the leaf phloem for redistribution to developing sinks, such as flowers, fruits and seed (Pate and Gunning, 1972; Lalonde *et al.*, 2003; Tegeder and Rentsch, 2010; Tegeder, 2012). In rice, even a young leaf plays a role as a supplier of remobilized nitrogen (Mae and Ohira, 1981). Therefore, there is a need for recycling of nitrogen and other solutes between the xylem and phloem that involves amino acid uptake from the xylem into the xylem parenchyma and transfer into the phloem (Tegeder and Rentsch, 2010; Okumoto and Pilot, 2011; Nagai *et al.*, 2013). In addition, within leaves there is another pathway for the retrieval of solutes from the xylem sap via the hydathodes. The hydathodes allow the excretion of xylem sap (guttation) from the leaf margins, where they are positioned close to the ends of the conducting vessels. Guttation is driven by root pressure (Dieffenbach *et al.*, 1980; Fisher *et al.*, 1997). Possible functions of guttation might be the refilling of the xylem vessels after embolism (Ewers *et al.*, 1997) and/or the exudation of toxic ions that are prevented from entering the mesophyll by, for example, the bundle sheath (Shatil-Cohen and Moshelion, 2012). In hydathodes, wall ingrowths typical of transfer cells are well developed in cells adjacent to the xylem (Maeda and Maedia, 1988), and may be regarded as a form of xylem parenchyma (Pate and Gunning, 1972). The hydathode functions as a selective reabsorption system with guttation fluid from the hydathode containing less K^+^ and NO_3_^−^, and no Pi, compared to xylem exudates in barley (Nagai *et al.*, 2013). Genes associated with the transport of a range of substances, including nitrate (Nazoa *et al.*, 2003), sucrose (Schulze *et al.*, 2000) and hexose (Schofield *et al.*, 2009), as well as a glutamine dumper protein (Pilot *et al.*, 2004; Pratelli *et al.*, 2010), are expressed in hydathodes and the composition of guttation fluid is complex (Singh and Singh, 2013).

The question then arises as to how much of the excess solutes that arrive in the xylem are simply transported to the phloem *via* the intervening cells (xylem and phloem parenchyma) and how much they are metabolized (including by the hydathodes). Tegeder and Rentsch (2010) have provided an overview of transport in xylem–phloem transfer, with Hsu and Tsay (2013) studying evidence for the roles of the amino acid transporters AtAAP2 and AtAAP6, and nitrate transporters. Enzymes of nitrogen and carbon metabolism are located in the vasculature and in the hydathodes. Glutamine synthetase (GS1) protein has been detected in companion cells and vascular parenchyma cells of senescing leaf blades of rice, where it may generate Gln for transport of nitrogen to sink tissues (Kamachi *et al.*, 1992; Sakurai *et al.*, 1996; Yamaya and Kusano, 2014). In barley leaves, GS has similar locations, being prominent in the bundle sheath, mestome sheath and xylem parenchyma (Leegood, 2008). NADH-glutamate synthase (NADH-GOGAT) protein was found to accumulate in vascular parenchyma cells and the mestome sheath cells of developing young rice leaves (Hayakawa *et al.*, 1994; Yamaya and Kusano, 2014). In the phloem parenchyma, an enzyme of carbohydrate metabolism, sucrose synthase, is also involved in the pathway of solutes to the phloem (Nolte and Koch, 1993). Cells surrounding the vascular system are enriched in enzymes that are not only key to photosynthesis in C_4_ plants, but also function in organic and amino acid metabolism in C_3_ plants (Hibberd and Quick 2002; Brown *et al.*, 2010). In Arabidopsis all four NADP-malic enzyme (NADP-ME) genes are expressed in or around the leaf vasculature and in the hydathodes (Gerrard Wheeler *et al.*, 2005). In barley leaves, NADP-ME protein is predominantly located within the xylem parenchyma (Leegood 2008). Both cytosolic and chloroplastic forms of pyruvate, orthophosphate dikinase (PPDK) accumulated preferentially in veins (Taylor *et al.*, 2010). Phospho*enol*pyruvate carboxykinase (PEPCK) protein is located within the the vascular tissues in a range of plants, such as grape, maize roots and leaves (Walker *et al.*, 1999), in cucumber phloem companion cells and vascular parenchyma (Chen *et al.*, 2004), in the vasculature of Arabidopsis (Chen *et al.*, 2004; Malone *et al.*, 2007; Brown *et al.*, 2010; Penfield *et al.*, 2012) and in Arabidopsis hydathodes (Penfield *et al.*, 2012). In addition, there is evidence that PEPCK participates in the metabolism of Asn in tissues involved in transport in pea, presumably by metabolizing the oxaloacetate that is generated from Asn via aspartate (Delgado-Alvarado *et al.*, 2007) and that cytosolic PPDK functions in nitrogen remobilization in senescing leaves (Taylor *et al.*, 2010) and in gluconeogenesis during seedling establishment (Eastmond *et al.*, 2015).

The aim of this study was to determine the role of metabolism, specifically of the ‘C_4_’ enzymes in rice leaves, in determining the amount and composition of guttation fluid and xylem sap in rice. This was done by determining how the amounts of transcripts and protein for these enzymes as well as the amount and composition of guttation fluid and xylem sap were influenced by N and by leaf development, and specifically what contribution is played by PEPCK to N recycling from the xylem in rice leaves.

## Materials and methods

### Plant material

*Oryza sativa* L. Indica cultivar IR72 seeds were sown on moist filter paper and incubated in sterile petri-dishes at 29 °C for 3 d then transferred to a growth chamber at a PPFD of 450 µmol m^−2^ s^−1^ with a 12h photoperiod (27 °C day, 24 ^o^C night, 60% humidity). For the first amino acid measurements of xylem sap and guttation fluid, seedlings were sown in trays containing Levington M3 compost (Scotts, Ipswich, UK) for a further 4 d. On day 7, guttation fluid and xylem sap was collected from the cut base of the primary leaf using a clean, sterile tip attached to a pipette, flash frozen in liquid nitrogen and stored at −80 °C. The entire experiment was repeated three times with new seedlings. All subsequent guttation fluid and xylem sap samples were collected by this method. Guttation fluid at the tip was collected immediately after the 12h dark period and for all subsequent experiments. The first xylem sap sample was collected 1h into the light period for all experiments. All samples subsequently detailed were subject to flash freezing in liquid nitrogen and subsequent storage at −80 °C. For all the subsequent feeding experiments, seedlings were sown on moist filter paper and incubated in sterile petri-dishes at 29 °C for 3 d. Each seedling was transferred to an individual compartment of a black, plastic seed tray filled with washed perlite and placed in a propagator tray partially filled with deionized water so that the roots of the seedlings were kept moist for a further 4 d. For subsequent feeding experiments water was removed from the trays and immediately replaced with the appropriate metabolite solution. There were no other nutrients provided for the seedlings other than those detailed in any of the subsequent feeding experiments.

For the quantitative RT-PCR experiment on day 6, one tray of 24 seedlings was incubated with either water, 10mM L-Asn, or 10mM L-Gln for 24h with propagator lids in place. All solutions in this and subsequent experiments were adjusted to pH 6.0–7.0. On day 7, primary leaves were harvested and aligned with their bases on a pre-cooled glass plate. A sterile surgical blade was used to cut leaves into successive 0.5cm sections which were pooled into sterile RNase-free Eppendorf tubes and frozen in liquid nitrogen. Leaf section samples were subsequently analysed by quantitative RT-PCR. The entire experiment was repeated three times with new seedlings. All leaf samples for RT-PCR and subsequent experiments were taken 2h into the light period.

For the Western immunoblotting experiment on day 6, one tray of 24 seedlings was each incubated with water, 10mM Asn, 10mM Gln, 10mM L-Ala and 10mM L-malic acid for 24h with propagator lids in place. On day 7, primary leaves were harvested and aligned with their bases on a pre-cooled glass plate and harvested as detailed for the quantitative RT-PCR experiment. In addition, the FW of each sample was recorded. Leaf section samples were subsequently analysed by Western-immunoblotting.

For amino acid and xylem exudate volume determinations plants were fed as detailed above for Western-immunoblotting. For the 3-mercaptopicolinic acid (MPA) feeding experiments seedlings were grown as described for the control (water) and 10mM Asn feeding. Trays of 24 seedlings were fed for 24h with either water (control), or water with 350 µM MPA, 10mM Asn, or 10mM Asn with 350 µM MPA. On day 7 guttation fluid was collected from each tray of 24 seedlings, pooled and flash frozen in liquid nitrogen. A clean razor blade and ruler was used to remove 1.5cm from the tip of each *in situ* seedling and the propagator lid replaced for 1h to prevent drying out. After 1h exuded xylem sap was collected and pooled for each treatment tray and frozen. A further 1cm was cut cleanly from each primary leaf and exuded xylem sap collected again after 1h. Finally, the remaining leaf was cut off at the base for all seedlings and xylem sap collected after 1h as previously described. The entire experiment was repeated three times with new seedlings.

Protein concentrations in xylem exudate were determined by the Bradford method (Bio-Rad, Hemel Hempstead, UK) using samples from control (water fed) plants as described above for the control/MPA feeding experiments. For the investigation into the effect of MPA feeding on amount of PEPCK protein, intact leaves were taken from control (water-fed) and MPA-fed plants as described above and subject to SDS-PAGE and immunoblotting as detailed below.

For the immunohistochemistry investigations seedlings were grown as previously described for compost. At 28 d, 2mm slices of the first 4mm of young leaf tips were harvested into fixative using at least ten leaves.

### Amino acid analysis

Amino acid analysis was performed using high performance liquid chromatography (HPLC). Guttation fluid samples were filtered directly through 0.20 µm Millipore filters whilst xylem sap samples were diluted with HPLC grade water prior to filtration. All samples (20 µl) were mixed with 200 µl of borate buffer (pH 10) and 30 µl of *ortho*-phthalaldehyde (OPA) for exactly 45s. A sample of 25 µl was then loaded after a further 60s onto a Luna C8 (250×4.6mm) column (Phenomenex, Macclesfield, UK) equilibrated with 25% (v/v) methanol, 75% (v/v) buffer [200mM Na acetate (pH 5.9), 1.5% (v/v) tetrahydrofuran]. Amino acids were eluted with a gradient of 25% (v/v) methanol, 75% (v/v) acetate buffer, to 90% (v/v) methanol, 10% (v/v) acetate buffer over 60min at a flow rate of 1.4ml min^−1^. The separated amino acids were detected by fluorescence using an excitation wavelength of 340nm and an emission wavelength of 455nm. Standard solutions of the amino acids were run under the same conditions to quantify each amino acid.

### RTq-PCR

Total RNA from pooled leaf sections was purified from 7-d-old *O. sativa* using an RNeasy Plant Mini Kit (Qiagen, Crawley, UK). RNA was treated with DNase (Sigma-Aldrich, Poole, UK), cleaned with 1:1 (v/v) phenol:chloroform, ethanol precipitated and air-dried. RNA quantity was determined spectrophotometrically and the quality of RNA was validated by agarose gel electrophoresis [1.5% (w/v) agarose]. A 2 µg aliquot of each total RNA sample was used in reverse transcription reactions to make cDNA strands using random hexamers as primer and a Bioscript RTq-PCR kit (Bioline, London, UK). cDNA was diluted 10-fold with sterile water.

Primers were designed by QuantPrime programme (http://www.quantprime.de/) (Arvidsson *et al.*, 2008) using an *O. sativa* Japonica database. Expression of target genes was normalized to U3, the small nucleolar RNA associated with protein 11 (*SnU3*-RNA). Target genes were *PEPCK3, 10, NADP-ME* and also genes for the *Glutamine Dumper1* (*GDU1*), *Glutamine synthetase* (*GS*) and *Asparaginase*. The sequences of primers used to detect these are listed in Supplementary Table S1 at *JXB* online. Primers to all genes except *GDU1* spanned an exon-exon junction further decreasing the possibility of amplification of genomic DNA. *SnU3*-RNA primers were selected from a range of reference gene primers after testing with RNA from all leaf sections. *SnU3*-RNA had a coefficient of variation of 28% under all conditions. All primer pairs for both reference and target genes were initially evaluated by checking PCR amplifications for every pair using at least five replicates. PCR amplifications from primer pairs that did not generate a single product and melt curves with a single uniform peak were rejected and new primers were selected. During analysis any amplification failing to meet this criterion was not included. A no-template control was analysed with all sample amplifications.

RTq-PCR was done using 1 µl of cDNA sample, 1 µl of primer mix (0.5 µM of each), 3 µl of water and 5 µl of 2× Sensimix (Quantace Sensimix NoRef Kit, Bioline, London, UK) following the manufacturer’s instructions using the RG6 000 (Corbett Research, UK). Parameters used were: 10min activation; followed by denaturation for 10s at 95 °C, annealing for 15s at 61 °C and extension for 25s at 72 °C for 45 cycles. The fluorescence intensity of SYBR green I was read and acquired at 72 °C after completion of the extension step of each cycle. Quantitation of individual transcripts was performed using the ‘Comparative Quantitation’ software supplied by Corbett Research for the Rotorgene. The mean efficiency of a group of cycling curves was calculated at the point that the cycling curves take off and was used to calculate a fold change according to the formula: fold change=efficiency^Ct1−Ct2^ where Ct1 and Ct2 were the take-off values of the cycling curves being compared. These fold-change values were expressed as the expression level relative to *SnU3*-RNA set to a value of 1 for each leaf section. SE values were calculated from the mean of two (technical) replicates for three independent experiments (biological replicates).

### SDS-PAGE and western immunoblotting

Pooled leaf sections (10–20mg) were homogenized in a mortar containing 15 volumes of ice-cold 200mM Bicine-KOH (pH 9.8), 50mM dithiothreitol (DTT), then clarified by centrifugation at 14 000 ×*g* for 5min. Supernatants were added to an equal volume of SDS/PAGE solubilization buffer [62.5mM Tris/HCl (pH 6.8), 10% (v/v) glycerol, 5% (w/v) SDS, 5% (v/v) 2-mercaptoethanol, 0.002% (w/v) bromophenol blue], placed at 100 °C for 3min, centrifuged at 14 000 ×*g* for 3min and supernatants analysed by SDS/PAGE.

SDS/PAGE was carried out using a 4.7% T/2.7% C stacking gel and a 7.5% T/2.7% C resolving gel. After electrophoresis, polypeptides were fixed in gels by immersion in 50% (v/v) methanol and 12% (v/v) acetic acid. Polypeptides were visualized by colloidal Coomassie Blue G-250 (Sigma-Aldrich, Poole, UK). For Western immunoblotting, transfer of polypeptides from an SDS/PAGE gel to Immunoblot PVDF membrane (Bio-Rad, Hemel Hempstead, UK) was done in a Bio-rad Tetra cell blotting apparatus. Immunoreactive polypeptides were visualized using a goat anti-rabbit IgG peroxidase-conjugated secondary antibody (Sigma-Aldrich Co. Ltd., Poole, UK) in conjunction with an enhanced chemiluminescence kit, and Hyperfilm ECL (GE Healthcare, Little Chalfont, UK) was used with an intensifying screen for up to 1min. For the various treatments on different Western immunoblots, adjustment to the same background brightness threshold was carried out using Photoshop.

### Immunohistochemistry

Thin slices of leaf tissue (2mm) were cut and immediately immersed in fixative [7.5mg ml^−1^ sucrose, 30mg ml^−1^ formaldehyde in 100mM NaH_2_PO_4_ (pH 7.2)] and placed under vacuum (25 °C, five times for 30min each) and then left overnight at 4 °C. Samples were fixed, embedded and mounted as described by Walker *et al.* (1997) using the indicator substrate 5-bromo-4-chloro-3-indolyl phosphate/nitroblue tetrazolium (BCIP/NBT) tablets (Sigma-Aldrich, Poole, UK).

### Antibodies

All antibodies were polyclonal and raised in rabbit. The antiserum specific for PEPC was raised against the enzyme purified from *Amaranthus edulis* L. (Dever *et al.*, 1995). Antiserum specific for PEPCK raised against the enzyme purified from cucumber (*Cucumis sativus* L.) (Walker *et al.*, 1995) was used in one of the immunolocalization studies. All other antibodies were affinity purified and derived from peptides designed to specific sequences in the corresponding enzymes in rice. See Supplementary Fig. S1 for details.

### Measurement of PEPCK activity

Material (50mg) was homogenized in a mortar containing five volumes of ice-cold 200mM Bicine-KOH (pH 9.8), 50mM dithiothreitol (DTT), then clarified by centrifugation at 14 000 ×*g* for 5min. The carboxylase activity of PEPCK was measured in the supernatant (Walker *et al.*, 2002). One unit of PEPCK activity produces 1 µmol product per min at 25 °C.

## Results

### Composition of xylem exudate and guttation fluid

**Fig. 1.**
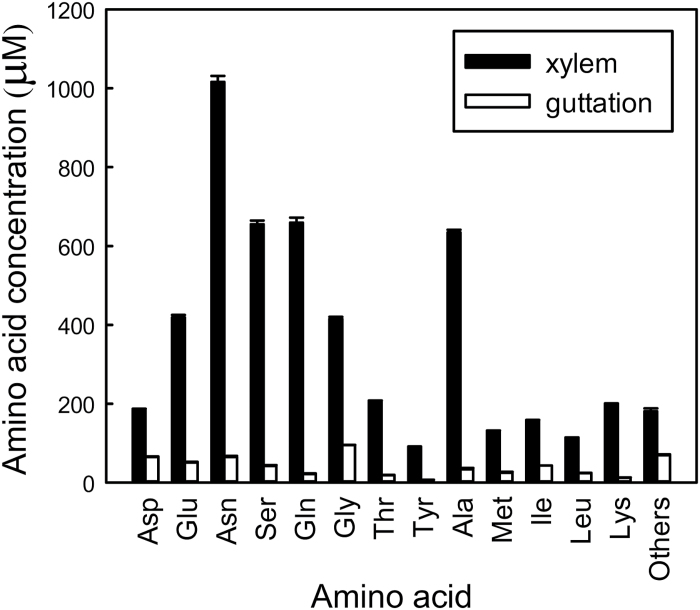
Amino acid concentrations of xylem exudate and guttation sap in 7-d-old first leaves of *O. sativa*. The xylem exudate and guttation fluid measurements are from the combined volume of 24 plants. Values are the mean ±SE from three independent experiments. All measurements of amino acid contents in the xylem exudate are significantly different at the 5% level when compared to the respective guttation sap.

Figure 1 shows the concentrations of amino acid and amides (hereafter collectively referred to as amino acids) of xylem exudates, sampled from the leaf base and guttation fluid in young leaves of intact rice plants. It shows that the composition of the guttation fluid differs markedly from the xylem sap and that there is reabsorption of N across the whole range of amino acids, with particularly marked reductions in the concentrations of Asn, Gln, Ser and Ala.

In addition to guttation fluid it was also possible to collect xylem exudate from cut leaves. There was no guttation or xylem sap exudation from detached leaves placed in water. Guttation and xylem sap exudation was collected from leaves still attached to the seedling.

**Fig. 2.**
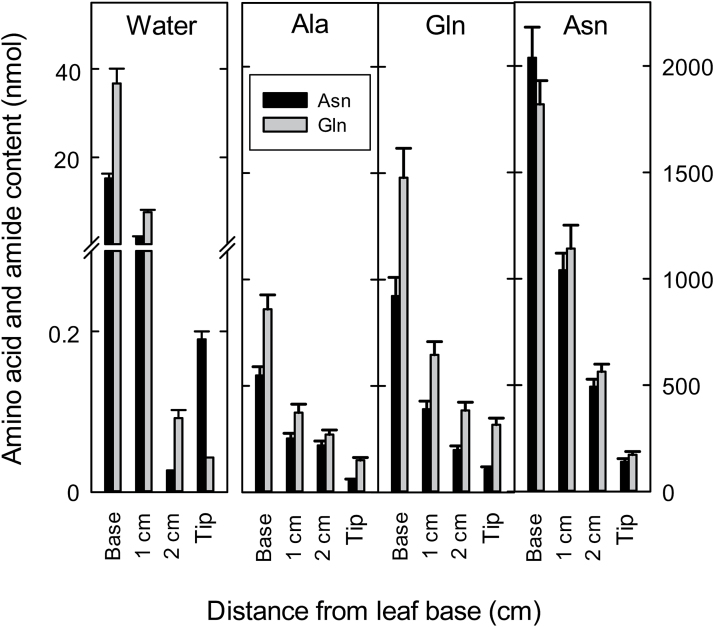
Amino acid and amide contents in guttation fluid at the tip (entire amount collected immediately after the 12h dark period) or xylem sap both at and various distances from the base (collected at 1h intervals in the light period) in 7-d-old primary leaves of *O. sativa* fed water (control), 10mM L-alanine, 10mM L-glutamine, or 10mM L-asparagine. The xylem exudate and guttation fluid measurements are from the combined volume of 24 plants. Values are the mean ±SE from three independent experiments. All xylem exudate and guttation sap amino acid contents are significantly different at the 5% level when compared to the control. Note break in Y-axis. Mean guttation and xylem exudate volumes (μl) were as follows: water, base to tip, 60, 11.5, 0.2, 41; alanine, 101, 58, 53, 135; glutamine, 112, 62, 45, 70; asparagine, 140, 111, 62, 103.

Figure 2 shows the contents of Asn and Gln in the xylem sap exuded from the leaf base, the cut leaf and in the guttation sap in young rice plants supplied with water, Asn, Gln and Ala. As in Fig. 1, there was a large decrease in Asn and Gln between the basal xylem exudate and the guttation fluid exuded from the tip of the leaf. Feeding with Asn induced the highest amounts of Asn and Gln in the xylem sap exuded from the leaf base. Supplying Gln and Ala increased the amounts of both Asn and Gln in the xylem exudate. Supply of Ala, Gln and Asn significantly increased the amounts of these amino acids in the guttation sap compared with supplying water (note the difference in Y-axis scales). However, for all treatments the amide content in the xylem was significantly lower than at the base of the leaf, and lower still in the guttation sap, indicating effective recycling of these substances even with the elevated xylem contents that resulted from supplying these substances to the root system. Note that although Fig. 1 shows concentrations, the remaining data are shown as amounts of amino acids because the volumes changed markedly. Ultimately it is the total amount of amino acids passing through the xylem to the leaf tip that relates to fluxes, whereas concentrations of amino acids in the xylem will tend to be regulated. Volume data are provided so that conversions can be made.

Table 1 shows the volumes of guttation fluid (at the tip) or of xylem sap exuded from cut leaves at varying distances from the base collected over a period of 1h. Of note is that guttation fluid was exuded more slowly than xylem exudate and the volume shown was the entire amount of guttation fluid at the beginning of the photoperiod from intact plants. It should be emphasized that the exudate and guttation fluid measurements are not directly comparable as exudation is an experimentally controlled process occurring in the light whereas guttation relies upon leaf processes occurring in the dark that cannot be experimentally manipulated. We checked that there was no contamination of xylem exudates with phloem sap by assessing protein concentration in the exudates. The mean protein concentration at the base and tip (guttation fluid) was 9.9 and 5.62ng μl^−1^ respectively, comparable to that in the xylem sap, but 10 000 times lower than in the phloem (Richardson *et al.*, 1982; Atkins *et al.*, 2011). In the control, amounts of xylem exudate decreased from the leaf base to the tip. In order to modify the composition of the xylem sap we supplied metabolites to the roots of intact plants. Supplying Ala, Gln and Asn greatly increased the volumes of xylem exudate and guttation fluid, from a doubling at the base to as much as a several hundred-fold increase at 2cm. By contrast, feeding malate (which was also taken up and led to a 4.5-fold increase in malate in the basal xylem exudate and a 14-fold increase in the guttation fluid) (Supplementary Table S2) had no significant effect on the volume except for a 3-fold increase in xylem exudate at 1cm (Table 1; *P*=0.038).

**Table 1.** Volumes of guttation fluid at the tip (entire amount collected) or xylem exudate both at and various distances from the base (collected after 1h) in 7-d-old first leaves of rice plants fed various compounds at a concentration of 10mM, or 350 µM MPA Values are the mean ±SE of three independent experiments of 24 plants.

**Distance from base (cm**)	**Mean volume of xylem exudate/guttation fluid per compound ±SE (µl**)						
	**Control**	**Malate**	**Ala**	**Gln**	**Asn**	**Control+MPA**	**Asn+MPA**
Base	60±4.04	71±8.90	101±8.69	112±11.00	140±14.29	61±4.06	130±12.90
1	11.5±0.76	33±4.41	58±5.29	62±6.11	111±11.26	20±2.52	92±8.51
2	0.2±0.06	0.4±0.12	53±4.58	45±5.13	62±7.06	21±2.60	95±10.02
Tip	41±2.08	62±6.69	135±11.37	70±8.02	103±10.49	67±4.36	96±8.54

### Changes in gene expression after feeding Asn and Gln

**Fig. 3.**
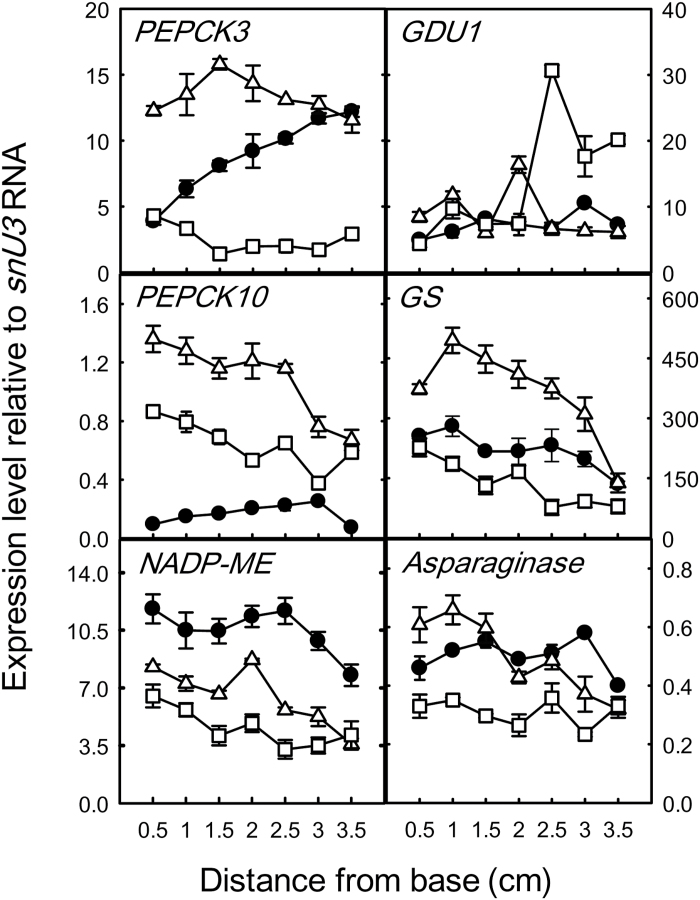
Relative expression of various genes compared to *snU3* RNA in 7-d-old control (●), asparagine-fed (∆) and glutamine-fed (□) first leaves of *O. sativa*. Results are expressed as means ±SE of three biological replicates.

Figure 3 shows the relative expression of selected genes compared to the control, small nuclear RNA U3 *(Os01g59500*) (*snU3*; Brown *et al.*, 2003), in leaves of young rice plants supplied with water, Asn or Gln. In leaves of plants supplied with water, expression of *PEPCK3 (Os03g15050*) increased from base to tip while expression of *PEPCK10* (*Os10g13700*) increased only slightly from the base until just below the tip (the numbers refer to the two PEPCK genes in rice that are found within chromosomes 3 and 10, respectively; Kawahara *et al.*, 2013). Expression of *NADP-ME* (*Os01g52500*) and *GS* (chloroplastic GS2; *Os04g56400*) tended to decrease from base to tip, while expression of *ASPARAGINASE* (*Os04g46370*) (Grant and Bevan, 1994) and *glutamine dumper 1* (*Os08g34700*) (*GDU1*, known to be expressed in the vascular tissues and in hydathodes of Arabidopsis; Pilot *et al.*, 2004) remained relatively constant. Supplying Asn generally increased expression of *PEPCK3*, *PEPCK10* and *GS*, although with overall declines of *PEPCK10* and *GS* along the leaf, it induced a decline in *ASPARAGINASE* along the leaf but had less effect on *GDU1*, and it decreased the expression of *NADP-ME* along the leaf. Supplying Gln decreased expression of *PEPCK3* (except at the base), *GS*, *NADP-ME* and *ASPARAGINASE*, but overall increased expression of PEPCK10 (although decreasing along the leaf) and increased expression of *GDU1* towards the leaf tip.

### Immunoblots for key enzymes

**Fig. 4.**
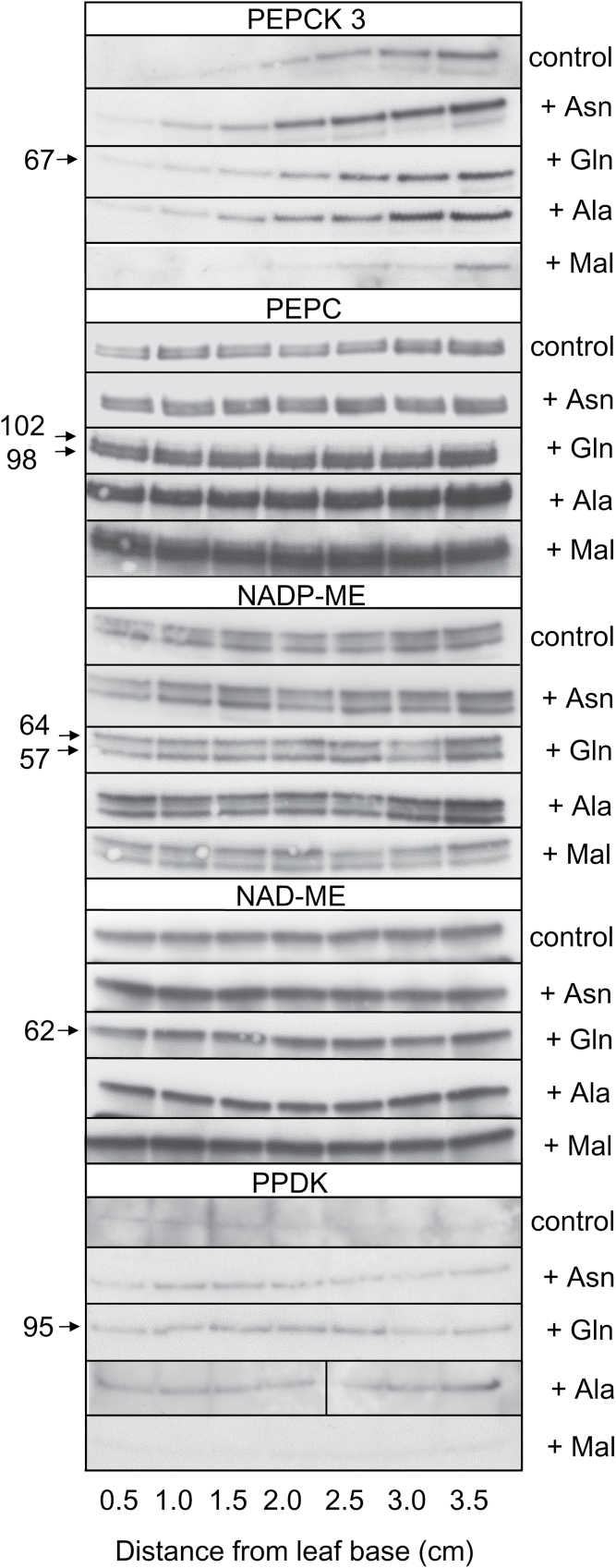
Immunoblots showing the effect of nitrogenous compounds or malate on the abundance of the PEPCK 3, PEPC, NADP-ME, NAD-ME and PPDK proteins in 0.5cm leaf sections taken from base to tip of the first leaf of 7-d-old leaves of *O. sativa*. Loadings on gels contained the soluble protein content of 0.33mg of FW of tissue. Numbers on the left hand Y-axis refer to molecular masses (kDa). Note that the image is constructed from different immunoblots.

Figure 4 shows that PEPCK3 protein increased from the base to the tip of the leaf. Feeding with Asn, Gln and Ala increased the amount of PEPCK3 protein, particularly in the middle region of the leaf (1.5–2.5cm from the base), while supplying malate decreased PEPCK3 in the middle region of the leaf. The amount of PEPC increased marginally from base to tip, and was increased along the entire length of the leaf by Gln, Ala and malate. The amounts of NAD-malic enzyme (NAD-ME) and two isoforms of NADP-ME were constant from the base to the tip of the leaf, with the amount of NADP-ME being slightly increased by the supply of Ala. The amount of PPDK was constant from the base to tip of the leaf but was slightly increased by supplying Asn and Gln, and notably by Ala at the leaf tip. Note that there was a good correspondence between transcripts (Fig. 3) and protein for control and Asn-fed PEPCK3, but not for Gln-fed PEPCK3 or for NADP-ME.

### Localization of PEPCK

**Fig. 5.**
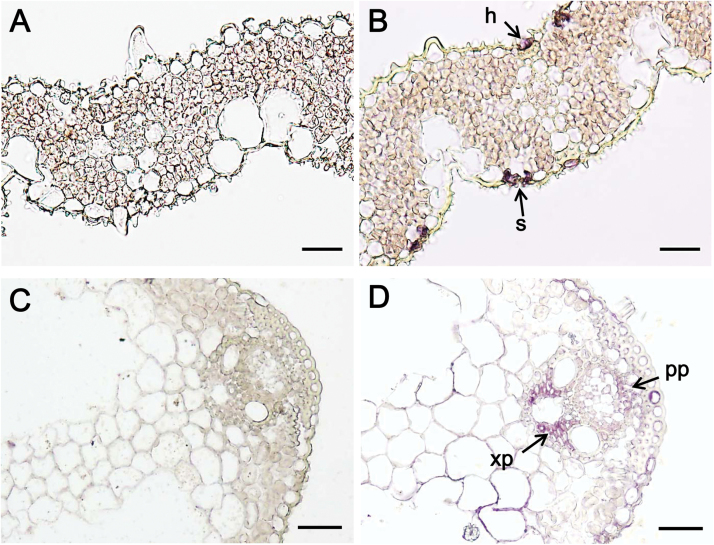
PEPCK is present in the stomata, s, hydathode, h, and vascular tissue (xylem parenchyma, xp; phloem parenchyma, pp). Transverse sections of rice after immunodiazostaining with pre-immune (A and C) and PEPCK serum (B and D). Serum used in panel B was a peptide antibody to the chromosome 3 isoform in rice. Serum used in panel D was a protein antibody to PEPCK in cucumber that was more sensitive to PEPCK located in the vascular tissue but less sensitive to PEPCK located in the stomata and hydathode of rice. Bars: 20 μm for A, B; 30 μm for C, D.

PEPCK was shown by immunolocalization to be present in the stomata, hydathodes and parenchyma cells close to the xylem and phloem (Fig. 5). Pre-immune controls showed no comparable staining in these cell types. A peptide antibody designed specifically to the chromosome 3 isoform of PEPCK in rice was used (Fig. 5B). This clearly showed staining in the hydathode and stomata, however staining in the vascular tissue was not as clear. This was due to the relatively weak expression of PEPCK in the vasculature and the high background coloration in the surrounding tissue. The peptide antibody was very specific because of affinity purification but as a consequence had to be used at a high concentration, leading to a high background coloration. We therefore used a different PEPCK antibody (Fig. 5D). This antibody was made using purified cucumber PEPCK as the antigen. Since it was a protein antibody (not affinity purified) it worked at a much more dilute concentration and unspecific background staining was largely eliminated. Staining of the vasculature was clearly visible, however this antibody was less reactive to PEPCK located in the stomata and hydathode of rice.

### Influence of decreased PEPCK activity on sap amount and composition

In order to test if PEPCK and its associated metabolism influenced the utilization and recycling of amino acids delivered in the xylem, we carried out two experiments to alter the activity of PEPCK. We supplied the intact plants with a specific inhibitor of PEPCK, 3-mercaptopicolinic acid (Leegood and ap Rees, 1978). In order to supply different compounds and MPA to seedlings it was also necessary to grow them in Perlite, rather than compost. One major effect of this change was that Asn was much less prominent as a solute transported in the xylem. Since PEPCK is implicated in Asn metabolism (Delgado *et al.*, 2007), we therefore did an additional treatment in which MPA was supplied to Asn-fed, as well as control plants. We checked, by immunoblotting, that supplying MPA for 24h did not affect the amount of PEPCK protein in rice leaves (Supplementary Fig. S2). Supplying MPA decreased xylem recycling in the leaf as there was an increase in the amount of amino acids, 2cm from the base and at the tip, either with or without an additional Asn supply. However, MPA made no difference to the total amino acids in the xylem exudate from the base of the leaf, with or without Asn, showing that there was no overall effect of MPA on the supply of amino acids from the roots (Table 2). Feeding MPA increased the volume of the xylem exudate and guttation fluid in the control leaves, except at the base, but had no additional effect when it was supplied with Asn (Table 1). Feeding MPA clearly inhibited Asn metabolism and resulted in an increase in amino acids and amides in the xylem. For full sets of data see Supplementary Tables S3, S4.

**Table 2.** Total amounts of amino acids in guttation fluid at the tip (entire amount collected) or xylem sap both at and various distances from the base (collected after 1h) in 7-d-old primary leaves of rice plants fed water (control) and water with 350 μM MPA or 10mM Asn (control) and Asn with 350 μM MPA Measurements are the mean ±SE of the combined volume from three independent measurements each of 24 plants. Mean guttation fluid and xylem exudate volumes (μl) required for conversion to μM are as follows: −MPA, base to tip, 60, 11.5, 0.2, 41; +MPA, 61, 20, 21, 67; –MPA+Asn, 140, 111, 62, 103; +MPA+Asn, 130, 92, 95, 96.

**Distance from base** **(cm)**	**Total amount of amino acids (nmol**)			
	**-MPA**	**+MPA**	**-MPA+Asn**	**+MPA+Asn**
Base	225±18	222±19	4900±340	5343±492
1	20.54±1.48	43.58±4.76	2813±241	2963±250
2	0.207±0.02	14.91±1.34	1280±86	2085±200
Tip	2.77±0.2	10.60±1.14	404±38	704±41

We did not find any activation-tagged lines in which PEPCK activity was reduced. However, we used an activation-tagged line of rice (in a different variety from the rest of the experiments) that had an elevated expression and amount of PEPCK (Hsing *et al.*, 2007). Table 3 shows that, compared with the wild type, leaves of the heterozygous and homozygous mutants had an increase in both PEPCK transcripts and up to a doubling of PEPCK activity. Increases in PEPCK activity in these plants led to a decrease in the total amount of amino acids and amides in the guttation fluid and a decrease in the guttation fluid volume.

**Table 3.** Characteristics of *Oryza sativa* L. Japonica cultivar Taichung wild-type and PEPCK transgenic lines (TRIM line M0035095) (Hsing *et al.*, 2007) Relative expression of PEPCK compared to glutaredoxin and PEPCK enzyme activity were measured in leaves. Copy number was determined to be one for the inserted gene. Guttation fluid was pooled from leaves of wild-type, heterozygous and homozygous plants, the volume measured and amino acid contents determined for each genotype. Values are the mean of three measurements from six plants ±SE. See Supplementary Tables S5–S7.

**Plant**	**Gene expression** **mean ±SE** **(% wild type)**	**Enzyme activity mean ±SE** **(µmol min** ^**−1**^ **g** ^**−1**^ **FWt**) **(% wild type)**	**Total amino** **acid content** **(nmol)**	**Guttation fluid volume** **(µl)**
Wild type	1.98±0.05 (100)	0.062±0.005 (100)	9.70±0.85	33±3
Heterozygote	3.01±0.22 (152)	0.091±0.006 (147)	4.92±0.46	27±3
Homozygote	5.71±0.42 (288)	0.135±0.019 (218)	2.51±0.13	13±1

## Discussion

The measurements of amino acids and amides in the guttation fluid and in the xylem exudates of cut leaves, when compared with the xylem exudate from the base of the shoot, provide striking evidence of the efficiency with which these nitrogenous compounds are reabsorbed from the xylem sap during its passage through the leaf. Feeding with Gln increased the contents of both Asn and Gln in the xylem exudate and also significantly increased the contents of these amino acids in the guttation sap perhaps indicating that Gln is not as effectively recycled as Asn.

**Fig. 6.**
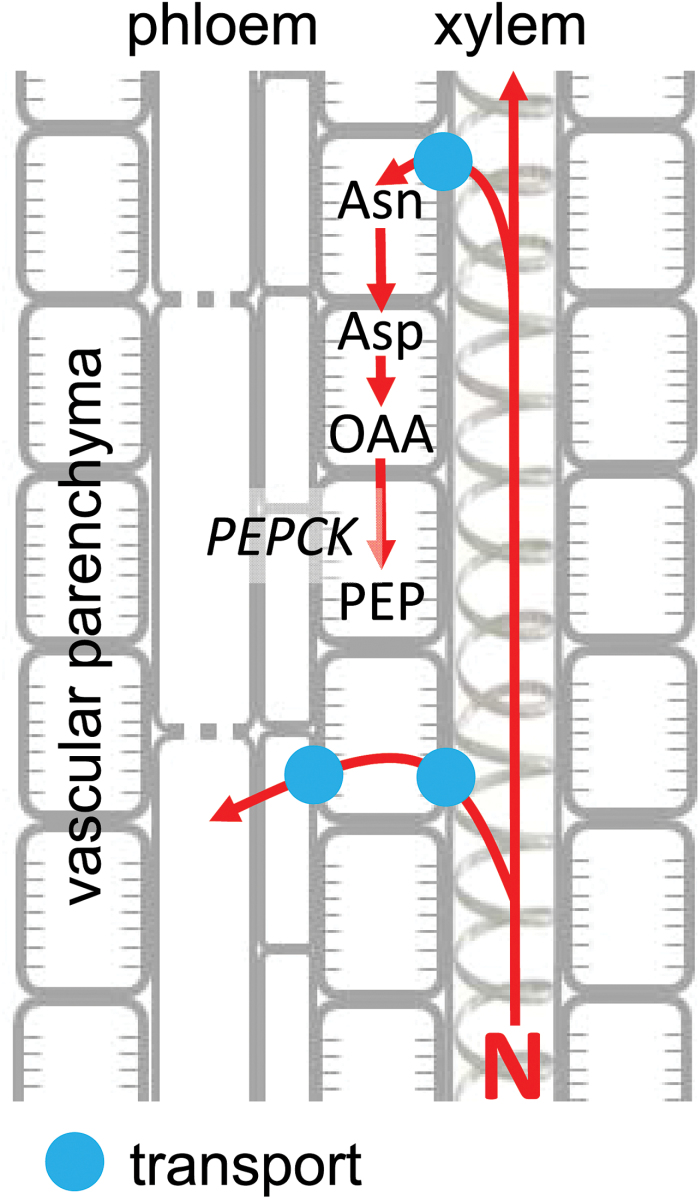
Schematic presentation of the roles of transport and the possible metabolic role of PEPCK in nitrogen recycling from the xylem. Transport involves unloading of amino acids and amides (N) from the xylem, passage through the vascular parenchyma between the xylem and phloem, and loading into the phloem. Metabolism involves similar transport processes in conjunction with metabolism in the vascular parenchyma. Specifically, the role of PEPCK would involve the metabolism of OAA (oxaloacetate) deriving from Asp and, ultimately, Asn. N released by Asn and Asp metabolism could then be also transported into the phloem.

Of course, amino acid recycling is essential to maintaining the nitrogen economy of the plant, but it then raises the question of whether this is achieved wholly by mechanisms involving intercellular transport or whether metabolism might also play a role. The fact that hydathodes are enriched in many enzymes of primary metabolism, as outlined in the introduction, led us to investigate whether metabolism might also be involved in N recycling. PEPCK is located in secretory tissues such as Arabidopsis hydathodes (Penfield *et al.*, 2012), and Arabidopsis nectaries and the stigma (Malone *et al.*, 2007), as well as in the xylem and phloem parenchyma cells associated with the vasculature, as outlined in the introduction and shown in Fig. 5. A survey of developmental changes in transcripts and protein for some of these key enzymes revealed that only transcripts for *PEPCK3* increased from the base to the leaf tip and that they were markedly increased by supplying Asn, while transcripts for *GLN DUMPER1* increased at the leaf tip when Gln was supplied. Supplying N also increased the amounts of protein of PEPCK and, to a much lesser extent, of PPDK. The pattern of *PEPCK3* transcript and PEPCK protein abundance in rice is similar to that observed for transcript abundance in the developing maize leaf (Li *et al.*, 2010). The observation that PEPCK protein is induced by a range of nitrogenous compounds (Chen *et al.*, 2004), and particularly by Asn in developing seeds (Walker *et al.*, 1999; Delgado-Alvarado *et al.*, 2007), suggests that it is involved in the metabolism of oxaloacetate deriving from Asn (via aspartate) in tissues that import or recycle nitrogen arriving as Asn (Delgado-Alvarado *et al.*, 2007). Thus in the hydathodes and vascular cells PEPCK may play a role in retrieving Asn from the xylem (Fig. 6).

Evidence for a role for PEPCK in xylem recycling in rice leaves was obtained by using MPA, a specific inhibitor of PEPCK in animals and plants (DiTullio *et al.*, 1974; Jomain-Baum *et al.*, 1976; Leegood and ap Rees, 1978; Wingler *et al.*, 1999) to show that inhibition of PEPCK resulted in an increase in amino acids in the guttation fluid as well as in the xylem exudate, except at the base. This finding was corroborated by using an activation-tagged rice line that had an increase in PEPCK activity showing that activation of PEPCK resulted in a decrease in amino acids in the guttation fluid. Taken together these results support the hypothesis that PEPCK is involved in N recycling. Furthermore, decreasing PEPCK increased the amount of xylem leaf exudate and guttation fluid whereas increasing PEPCK decreased the amount of guttation fluid. The simplest interpretation is that altering the efficiency of this recycling feeds back on xylem hydraulic conductivity.

There is previous evidence that transfer of N from the xylem to phloem involves metabolism as well as direct transfer of amino acids. Sharkey and Pate (1975) fed ^14^C-labelled amino compounds singly to fruiting shoots of white lupin through the transpiration stream and measured the distribution of ^14^C in solutes of the phloem sap. The carbon of Asn, the major nitrogenous solute of phloem, was derived from five amino compounds in the xylem. Although the most important of these transfers was the direct throughput of Asn from xylem to phloem, significant amounts of carbon in phloem Asn were also exchanged from aspartate, glutamate, Gln and γ-aminobutyrate supplied to the xylem. On the other hand, carbon in aspartate and glutamate in the phloem was derived from a wide number of xylem amino acids, with little direct transfer of either amino acid from the xylem to the phloem (Sharkey and Pate, 1975). Differences between the xylem and phloem sap composition of maize leaves might also have a similar explanation (Martin *et al.*, 2006).

Why metabolism is involved in xylem recycling remains an open question. One possibility is that metabolism is acting as a sink to promote N transfer from the xylem, another is that metabolism reconfigures the N profile to suit the immediate needs of the phloem, for example, to supply seeds (Sharkey and Pate, 1975) or developing tissues. If PEPCK were involved, then the pathway is likely to involve the release of ammonia from Asn, which could then be reassimilated via GS and GOGAT, enzymes which are present in the vasculature (Kamachi *et al.*, 1992; Hayakawa *et al.*, 1994; Sakurai *et al.*, 1996).

The other important finding from these studies was that the various experimental treatments modulated the amount of guttation and xylem exudate, suggesting that the amount of amino acids and amides in the xylem exudate modulated the hydraulic conductivity of the xylem and that this might change along the length of the leaf. It should be noted that malate had a much smaller effect, suggesting that N could be a key factor. Water relations and vascular metabolism and transport are thus intimately linked. An excess of amino acids in the xylem may be the signal involved in modulating xylem hydraulic conductivity. There is already evidence that nutritional information can be translated into a hydraulic response, whereby a plant’s transpiration, stomatal conductance and root hydraulic conductivity can be influenced by the supply of certain mineral nutrients (Clarkson *et al.*, 2000). The xylem is a finely regulated water transport system (Nardini *et al.*, 2011). Vascular bundle-sheath cells have been hypothesized to control the solute and hydraulic conductivity of the leaf vascular system (Fricke 2002), with the latter altered via the specific activity of bundle sheath cell aquaporins (Shatil-Cohen *et al.*, 2011). It is also established that water-transporting aquaporins are regulated in response to N (Gaspar *et al.*, 2003; Loqué *et al.*, 2005, Hacke *et al.*, 2010), probably by phosphorylation that is mediated both by N (Engelsberger and Schulze, 2012) and, specifically in the leaf veins, by light (Prado *et al.*, 2013).

The above findings allow us to propose the following hypotheses: (i) both metabolism and transport are involved in xylem recycling and (ii) excess N is a signal involved in modulating xylem hydraulic conductivity, whether in the roots or shoots. Thus feeding N increases hydraulic conductivity, and feeding MPA inhibits Asn metabolism and results in an increase in amino acids and amides in the xylem. This also appears to increase conductivity. These observations are not restricted to rice and we have made similar findings in both barley and maize (in which PEPCK also functions in the bundle sheath in C_4_ photosynthesis).

## Supplementary data

Supplementary data can be found at *JXB* online.

Fig. S1. Immunoblot showing the difference in specificity between antibodies specific to the chromosome 3 and the chromosome 10 isoform of PEPCK.

Fig. S2. Western immunoblot of PEPCK3 in MPA or water fed leaves.

Table S1. Sequences of primers used for gene expression.

Table S2. Concentration of malate in guttation fluid and xylem exudate in water- or malate-fed plants.

Table S3. Amounts of amino acids in guttation fluid and xylem exudate in plants fed water or water+MPA.

Table S4. Amounts of amino acids in guttation fluid and xylem exudate in plants fed Asn or Asn+MPA.

Table S5. Sequences of primers used for genotyping.

Table S6. Sequences of primers used for copy number determination.

Table S7. Sequences of primers used for PEPCK3 gene expression.

Supplementary Data

## Abbreviations:

GOGAT: glutamate synthase
GS: glutamine synthetase
MPA: 3-mercaptopicolinic acid
NADP-ME: NADP-malic enzyme
PEPCK: phosphoenolpyruvate carboxykinase
PPDK: pyruvate, orthophosphate dikinase.
