# Supplementary material for: Nitrogen recycling from the xylem in rice leaves: dependence upon metabolism and associated changes in xylem hydraulics
Source: J Exp Bot. 2016 Apr 6;67(9):2901–11. doi: 10.1093/jxb/erw132 (PMC4861031; doi:10.1093/jxb/erw132)
Supplement: Supplementary Data [file supp_67_9_2901__index.html]

Nitrogen recycling from the xylem in rice leaves: dependence upon metabolism and associated changes in xylem hydraulics — Nitrogen recycling from the xylem in rice leaves: dependence upon metabolism and associated changes in xylem hydraulics — Supplementary Data 

# Nitrogen recycling from the xylem in rice leaves: dependence upon metabolism and associated changes in xylem hydraulics

## Supplementary Data

Data files

- supplementary\_figures\_S1\_S2\_tables\_S1\_S7.pdf - Supplementary Data
