# Supplementary material for: Nitrogen recycling from the xylem in rice leaves: dependence upon metabolism and associated changes in xylem hydraulics
Source: J Exp Bot. 2016 Apr 6;67(9):2901–11. doi: 10.1093/jxb/erw132 (PMC4861031; doi:10.1093/jxb/erw132)
Supplement: Supplementary Data [file supp_erw132_supplementary_figures_S1_S2_tables_S1_S7.pdf]

Nitrogen Recycling from the Xylem in Rice Leaves: Dependence upon Metabolism and Associated Changes in Xylem Hydraulics

Karen J Bailey and Richard C Leegood

Antibodies used in Western Immunoblotting and Immunolocalisation

All antibodies except for PEPC (derived from purified *Amaranthus edulis L.* protein) (Dever *et al.* 1995) and PEPCK (derived from purified *Cucumis sativus L.* protein) (Walker *et al.* 1995) were affinity purified and derived from peptides designed to specific sequences in the corresponding enzymes in rice. Two peptide antibodies were designed to the two isoforms of PEPCK in rice, chromosome 3 and chromosome 10 isoforms. Each antibody specifically detected either the chromosome 3 isoform or the chromosome 10 isoform. The peptide antibody to the chromosome 3 isoform of PEPCK specifically detected this isoform due to the targeting of a string of amino acids present only in the longer chromosome 3 protein. The affinity purified antibodies by nature of the affinity purification elution process were more dilute in their final form compared to the PEPC and *Cucumis sativus*. PEPCK final form antibodies.

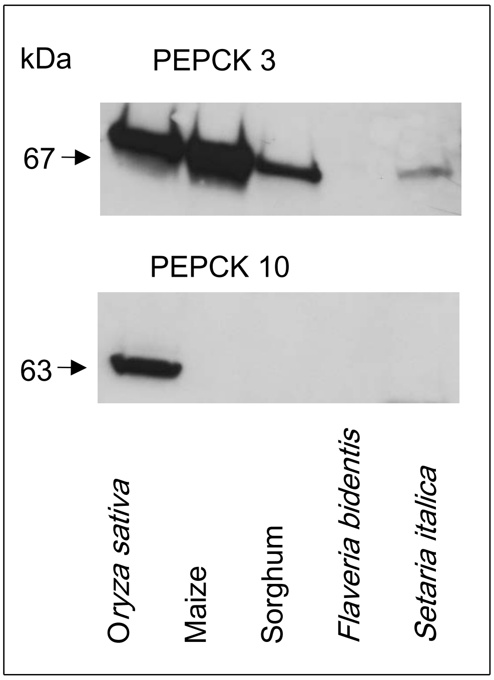

Supplementary Fig. S1. Immunoblot showing the difference in specificity between the peptide antibodies of rice specific to the chromosome 3 and the chromosome 10 isoform of PEPCK. The fifth leaf of 42-d-old plants were used except for *S. italic* and *F. bidentis* in which case 14-d-old leaves were used. Loadings on the gel contained the soluble protein

content of 2.0 mg of FW of tissue. For specificity of *Cucumis sativus L.* PEPCK please see Walker *et al*. (1995).

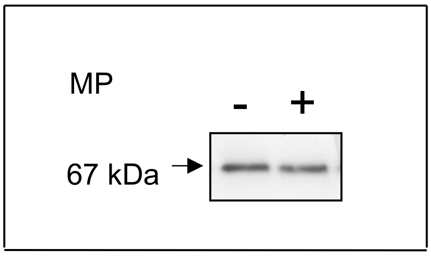

Supplementary Fig. S2. Immunoblot showing the effect of 3-mercaptopicolinic acid on the abundance of the PEPCK 3 protein in 7-d-old first leaves of *O. sativa* plants fed for 24 hours. Control plants were fed with water. Treated plants were fed with water containing 350 mM MPA. Loadings on gels contained the soluble protein content of 1 mg of FW of tissue.

Supplementary Table S1. *Primers used for gene expression.*

Sequences of primers used for relative gene expression experiment described in Figure 3. Target genes GDU1, ASPARAGINASE, GS, PEPCK3, PEPCK10 and NADP-ME were normalised to the small nucleolar RNA associated with protein 11 (SnU3-RNA*).*

Gene	Chromosome	Forward Primer	Reverse Primer
SnU3-RNA	Os01g59500	ACATGGCCTTGCAGAAAGAGTTG	GCCATTCACATCATCGCTTCCG
GDU1	Os08g34700	TTGTGGAACACAGAACGCACAG	TTTGGCGGCAACAAAGAACTCC
ASPARAGINASE	Os04g46370	AGCCCAGATGAGAATGGTGAAAC	CCGATTTCCATTGTGGTCCCATC
GS	Os04g56400	TGGGTGCCACACAAACTACAGC	GCAAGTCATGGCGAAGTGATAGG
PEPCK3	Os03g15050	AGTGGCTACACTGCTCTGGTTG	TGGAGCATGATGAACGCTGCAC
PEPCK10	Os10g13700	TCAGTGGCTACACTGGCATTGGTC	TAGCCTGTGGCTCCTTGATACC
NADP-ME	Os01g52500	TGACATGCTTCTTGCAGCTTCG	GGCCCTTGTCGAAATTCTCTTGTG

Supplementary Table S2. *Concentration of malate in guttation sap and xylem exudate in 7-d-old rice plants supplied with water (control) or 10 mM L-malate. Values are the mean ± SE of three separate sets of 24 plants.*

Treatment	Guttation sap/Xylem exudate	Malate concentration (µM)
Control	Guttation sap	1.76 ± 0.09
	Xylem exudate	40.00 ± 0.50
Malate	Guttation sap	24.53 ± 0.13
	Xylem exudate	181 ± 3

Supplementary Table S3. *Amounts of amino acids in guttation fluid at the tip and xylem exudate both at and various distances from the base in 7-d-old rice plants grown on perlite and supplied with water (control) and water with 350 μM MPA. Values are the mean ± SE of three separate sets of 24 plants. Mean guttation and xylem exudate volumes (μl) required for conversion to μM are as follows: control, base to tip; 60, 11.5, 0.2, 41. MPA, 61, 20, 21, 67.*

	Amount of amino acids (nmol)							
	Base		1.0 cm		2.0 cm		Tip	
	Control	MPA	Control	MPA	Control	MPA	Control	MPA
Aspartate	6.12 ± 0.54	3.66 ± 0.26	0.49 ± 0.03	0.99 ± 0.08	0.003 ± 0	1.15 ± 0.11	0.212 ± 0	0.93 ± 0.08
Glutamic Acid	3.59 ± 0.25	2.35 ± 0.18	0.41 ± 0.03	0.84 ± 0.05	0.002 ± 0	0.82 ± 0.09	0.360 ± 0.02	0.25 ± 0.15
Asparagine	15.24 ± 1.08	27.27 ± 2.32	1.97 ± 0.18	7.20 ± 0.48	0.027 ± 0	1.72 ± 0.16	0.197 ± 0.01	0.54 ± 0.06
Histidine	9.96 ± 0.60	3.78 ± 0.30	0.14 ± 0.01	0.32 ± 0.01	0.001 ± 0	0.35 ± 0.03	0.103 ± 0.01	0.40 ± 0.03
Serine	24.06 ± 1.38	19.76 ± 1.34	1.24 ± 0.09	1.34 ± 0.09	0.009 ± 0	0.88 ± 0.07	0.311 ± 0.03	1.74 ± 0.12
Glutamine	36.72 ± 3.36	88.02 ± 7.56	7.61 ± 0.52	22.56 ±2.28	0.092 ± 0.01	1.74 ± 0.13	0.043 ± 0	0.32 ± 0.03
Arginine	5.80 ± 0.31	4.84 ± 0.52	0.25 ± 0.01	0.71 ± 0.06	0.002 ± 0	0.53 ± 0.04	0.142 ± 0.01	0.19 ± 0.02
Glycine	12.12 ± 1.08	10.80 ± 1.10	1.07 ± 0.04	1.65 ± 0.18	0.009 ± 0	1.00 ± 0.12	0.378 ± 0.03	3.48 ± 0.31
Threonine	16.74 ± 1.98	6.59 ± 0.54	0.47 ± 0.05	1.36 ± 0.15	0.006 ± 0	1.41 ± 0.16	0.178 ± 0.02	0.67 ± 0.10
Tyrosine	8.88 ± 0.54	3.88 ± 0.32	0.41 ± 0.02	0.53 ± 0.05	0.003 ± 0	0.25 ± 0.02	0.037 ± 0	0.23 ± 0.02
Alanine	6.72 ± 0.78	5.52 ± 0.54	0.82 ± 0.05	1.00 ± 0.06	0.006 ± 0	0.70 ± 0.06	0.282 ± 0.02	0.41 ± 0.05
GABA	8.34 ± 0.02	3.76 ± 0.37	0.44 ± 0.03	0.66 ±0.06	0.005 ± 0	0.53 ± 0.05	0.029 ± 0	0.03 ± 0
Tryptophan	0.35 ± 0.02	3.46 ± 0.27	0.04 ± 0	0.24 ± 0.02	0.001 ± 0	0.21 ± 0	0.039 ± 0	0.06 ± 0.01
Methionine	1.14 ± 0.07	1.13 ± 0.10	0.14 ± 0.01	0.18 ± 0.02	0.001 ± 0	0.13 ± 0.01	0.024 ± 0	0.02 ± 0
Valine	26.82 ± 2.28	12.32 ± 1.10	1.81 ± 0.13	0.80 ± 0.07	0.017 ± 0	1.15 ± 0.12	0.157 ± 0.01	0.48 ± 0.05
Phenylalanine	2.49 ± 0.13	2.30 ± 0.26	0.21 ± 0.01	0.33 ± 0.36	0.002 ± 0	0.39 ± 0.02	0.057 ± 0	0.17 ± 0.01
Isoleucine	11.70 ± 0.96	2.26 ± 0.22	1.05 ± 0.08	0.49 ± 0.04	0.012 ± 0	0.28 ± 0.03	n.d.	0.33 ± 0.02
Leucine	11.22 ± 0.48	12.26 ± 1.10	1.18 ± 0.13	1.75 ±0.16	0.006 ± 0	1.00 ± 0.07	0.130 ± 0.01	0.21 ± 0.02
Lysine	17.28 ±1.86	8.30 ± 0.73	0.80 ± 0.05	0.65 ± 0.51	0.004 ± 0	0.66 ± 0.05	0.089 ± 0.0	0.12 ± 0.07
Σ amino acids	225 ± 18	222 ± 19	20.54 ± 1.48	43.58 ± 4.76	0.2069 ± 0.02	14.91 ± 1.34	2.77 ± 0.20	10.60 ± 1.14

Supplementary Table S4. *Amounts of amino acids in guttation fluid at the tip and xylem exudate both at and various distances from the base in 7-d-old rice plants grown on perlite and supplied with 10 mM Asn and Asn with 350 µM MPA. Values are the mean ± SE of three separate sets of 24 plants. Mean guttation and xylem exudate volumes (µl) required for conversion to µM are as follows: Asn, base to tip; 140, 111, 62, 103. Asn +MPA, 130, 92, 95, 96.*

	Amount of amino acids (nmol)							
	Base		1.0 cm		2.0 cm		Tip	
	Asn	MPA	Asn	MPA	Asn	MPA	Asn	MPA
Aspartate	28.42 ± 3.08	33.15 ± 2.86	29.64 ± 1.44	25.67 ± 2.02	6.45 ± 0.68	17.86 ± 1.99	4.65 ± 0.32	10.85 ± 0.77
Glutamic Acid	26.60 ± 2.38	23.40 ± 2.47	24.20 ± 1.11	17.11 ± 27.33	9.49 ± 0.87	15.30 ± 0.76	9.41 ± 1.03	9.89 ± 0.48
Asparagine	2039 ± 143	2377 ± 217	1040 ± 80.03	1110 ± 163	493 ± 34.84	832 ± 84.08	140 ± 14.52	227 ± 23.14
Histidine	30.24 ± 0.98	23.66 ± 2.21	17.98 ± 1.55	15.18 ± 14.11	4.92 ± 0.41	11.97 ± 1.14	2.08 ± 0.20	5.93 ± 0.50
Serine	199 ± 16.94	168 ± 14.69	102 ± 7.99	145 ± 95.21	40.24 ± 0.43	76.10 ± 6.84	15.97 ± 1.65	31.10 ± 2.69
Glutamine	1820 ± 111	2093 ± 202	1142 ± 110	1155 ± 609	564 ± 35.07	794 ± 77.05	173 ± 14.83	305 ± 3.17
Arginine	55.58 ± 3.36	40.82 ± 2.86	34.41 ± 2.44	30.54 ± 10.22	8.56 ± 0.68	26.41 ± 2.38	3.18 ± 0.23	8.99 ± 0.85
Glycine	117 ± 10.18	71.89 ± 4.03	51.95 ± 3.11	127 ± 70.55	19.59 ± 1.74	47.22 ± 4.09	7.16 ± 0.57	16.99 ± 1.34
Threonine	83.16 ± 8.82	65.65 ± 5.85	36.30 ± 3.77	41.31 ± 28.77	8.00 ± 0.87	19.10 ± 2.38	7.34 ± 0.60	10.94 ± 1.15
Tyrosine	23.66 ± 1.54	27.56 ± 1.43	16.32 ± 1.22	19.96 ± 18.76	7.19 ± 0.62	15.58 ± 1.62	4.33 ± 0.40	7.09 ± 0.54
Alanine	157 ± 12.46	123 ± 9.88	90.80 ± 8.10	83.26 ± 40.21	37.20 ± 2.79	63.56 ± 5.51	9.06 ± 0.81	21.50 ± 1.73
GABA	27.16 ± 2.94	20.93 ± 1.56	16.54 ± 1.78	14.08 ± 22.45	6.82 ± 0.50	11.50 ± 0.95	1.52 ± 0.14	2.70 ± 0.17
Tryptophan	10.84 ± 0.73	37.31 ± 4.03	12.77 ± 1.00	20.5 2± 0	0.13 ± 0.01	1.07 ± 0.11	n.d.	0.48 ± 0.05
Methionine	8.64 ± 0.60	9.05 ± 0.94	11.21 ± 0.89	5.79 ± 4.22	2.10 ± 0.25	6.96 ± 0.05	0.94 ± 0.01	2.18 ± 0.18
Valine	92.68 ± 7.14	70.59 ± 6.89	65.93 ± 6.88	48.39 ± 81.17	26.85 ± 2.36	47.98 ± 3.90	12.26 ± 1.24	18.05 ± 1.54
Phenylalanine	26.32 ± 1.54	21.71 ± 1.95	21.98 ± 1.33	14.26 ± 0	5.59 ± 0.61	11.21 ± 1.05	n.d.	1.33 ± 0.12
Isoleucine	16.94 ± 1.26	16.12 ± 1.69	9.61 ± 0.62	9.84 ± 47.81	3.83 ± 0.32	11.40 ± 0.76	1.61 ± 0.17	3.22 ± 0.39
Leucine	77.70 ± 8.54	79.95 ± 6.63	52.06 ± 4.66	50.88 ± 33.53	21.14 ± 1.74	48.17 ± 2.76	7.63 ± 0.82	13.63 ± 1.15
Lysine	59.92 ± 3.64	40.43 ± 3.64	38.41 ± 3.55	28.98 ±25.89	15.13 ± 1.61	27.55 ± 2.95	3.98 ± 0.33	6.94 ± 0.62
Σ amino acids	4900 ± 340	5343 ± 492	2813 ± 241	2963 ± 250	1280 ± 86	2085 ± 200	404 ± 38	704 ± 41

Transgenic plant isolation and copy number analysis

Primary isolation of transgenic plants. T_2 seeds of TRIM line M0035095 (Chromosome Os03g15050; supplied by the Institute of Plant and Microbial Biology, Academia Sinica, Taipei, Taiwan) were grown in compost as described in Materials and Methods. At 5 weeks the fifth leaf from the first tiller was harvested. DNA was extracted using a DNeasy Plant Mini Kit (Qiagen, Crawley, UK). 11.5 µl of DNA was mixed with 12.5 µl of BioMix Red (Bioline, London, UK) and 0.5 µM of each primer (see table below). PCR was done at 95°C for 5 min; then 95°C (15 s), 57°C (2 min), 72°C (1min 30 s), for 35 cycles and 72°C for 5 min. Plants were genotyped according to product fragments obtained on a 1% (w/v) agarose ethidium bromide stained gel and the ratio of each genotype noted. Seed was collected from each plant genotype and stored for further use.

Supplementary Table S5. *Primers used for genotyping.*

Sequences of primers used for genotyping of T_2 (above) and T_3 (Table 3) plants.

Gene	Left Primer	Right Primer	Border Primer
PEPCK 3 TRIM line M0035095	TACGATTTACGACCCGGTTC	TCCTGTTGAAAAGCAGGAGC	ACTCATGGCGATCTCTTACC

PCR of T_3 plants (from single selfed heterozygous T_2 transgenic plants) used in amino acid and guttation fluid measurements. DNA was extracted from 7-d-old plants grown and analysed as described above for T_2 seedlings. Amino acid analysis was done as detailed in Materials and Methods on guttation fluid from pooled plants confirmed by PCR to be wild-type, heterozygote or homozygote. Copy number was determined from the isolated DNA (IDna Genetics Ltd., The John Innes Centre, Norwich) using methodology detailed in Bartlett *et al*., (2008) and standard hygromycin gene primers (Table S6).

Supplementary Table S6. *Primers used for copy number determination.*

Sequences of primers used for copy number determination of plants described in Table 3.

Gene	HvHygP Probe Primer	HvHygF3 Forward Primer	HvHygR2 Reverse Primer
Hyg	Fam-CAGCGGTCATTGACTGGAGCGAGG-tamra	GGATTTCGGCTCCAACAATG	TATTGGGAATCCCCGAACATC

RTq-PCR. Quantitative PCR was done as detailed in the main Materials and Methods section using RNA from the transgenic TRIM line rice plants. Expression of the target gene *PEPCK3* was normalized to *Glutaredoxin*. The sequences of primers used are shown in Table S7.

Supplementary Table S7. *Primers used for gene expression.*

Sequences of primers used for relative gene expression described in Table 3. Target gene PEPCK3 was normalised to the Glutaredoxin gene.

Gene	Chromosome	Forward Primer	Reverse Primer
Glutaredoxin	Os04g42930	TGTTCGAGCAGCTTGGAGCAAC	ACTGCAGCTCAGATCCATCACTC
PEPCK3	Os03g15050	AGTGGCTACACTGCTCTGGTTG	TGGAGCATGATGAACGCTGCAC
